# Development and Validation of an Artificial Intelligence Model for Small Bowel Capsule Endoscopy Video Review

**DOI:** 10.1001/jamanetworkopen.2022.21992

**Published:** 2022-07-14

**Authors:** Xia Xie, Yu-Feng Xiao, Xiao-Yan Zhao, Jian-Jun Li, Qiang-Qiang Yang, Xue Peng, Xu-Biao Nie, Jian-Yun Zhou, Yong-Bing Zhao, Huan Yang, Xi Liu, En Liu, Yu-Yang Chen, Yuan-Yuan Zhou, Chao-Qiang Fan, Jian-Ying Bai, Hui Lin, Anastasios Koulaouzidis, Shi-Ming Yang

**Affiliations:** 1 Department of Gastroenterology, The Second Affiliated Hospital, the Third Military Medical University, Chongqing, China; 2 Department of Epidemiology, the Third Military Medical University, Chongqing, China; 3 Department of Public Health, Pomeranian Medical University, Szczecin, Poland

## Abstract

**Importance:**

Reading small bowel capsule endoscopy (SBCE) videos is a tedious task for clinicians, and a new method should be applied to solve the situation.

**Objectives:**

To develop and evaluate the performance of a convolutional neural network algorithm for SBCE video review in real-life clinical care.

**Design, Setting, and Participants:**

In this multicenter, retrospective diagnostic study, a deep learning neural network (SmartScan) was trained and validated for the SBCE video review. A total of 2927 SBCE examinations from 29 medical centers were used to train SmartScan to detect 17 types of CE structured terminology (CEST) findings from January 1, 2019, to June 30, 2020. SmartScan was later validated with conventional reading (CR) and SmartScan-assisted reading (SSAR) in 2898 SBCE examinations collected from 22 medical centers. Data analysis was performed from January 25 to December 31, 2021.

**Exposure:**

An artificial intelligence–based tool for interpreting clinical images of SBCE.

**Main Outcomes and Measures:**

The detection rate and efficiency of CEST findings detected by SSAR and CR were compared.

**Results:**

A total of 5825 SBCE examinations were retrospectively collected; 2898 examinations (1765 male participants [60.9%]; mean [SD] age, 49.8 [15.5] years) were included in the validation phase. From a total of 6084 CEST-classified SB findings, SSAR detected 5834 findings (95.9%; 95% CI, 95.4%-96.4%), significantly higher than CR, which detected 4630 findings (76.1%; 95% CI, 75.0%-77.2%). SmartScan-assisted reading achieved a higher per-patient detection rate (79.3% [2298 of 2898]) for CEST findings compared with CR (70.7% [2048 of 2298]; 95% CI, 69.0%-72.3%). With SSAR, the mean (SD) number of images (per SBCE video) requiring review was reduced to 779.2 (337.2) compared with 27 910.8 (12 882.9) with CR, for a mean (SD) reduction rate of 96.1% (4.3%). The mean (SD) reading time with SSAR was shortened to 5.4 (1.5) minutes compared with CR (51.4 [11.6] minutes), for a mean (SD) reduction rate of 89.3% (3.1%).

**Conclusions and Relevance:**

This study suggests that a convolutional neural network–based algorithm is associated with an increased detection rate of SBCE findings and reduced SBCE video reading time.

## Key Points

**Question:**

Can artificial intelligence be applied in video review of small bowel capsule endoscopy (SBCE)?

**Findings:**

In this diagnostic study of 5825 patients, a convolutional neural network solution was developed based on CE structured terminology (CEST) to allow a standardized computer-aided detection (CADe) approach. The convolutional neural network was associated with an increased detection rate of SB findings and reduced SBCE video reading times.

**Meaning:**

This study suggests that a well-structured CADe algorithm, based on CEST, may change the human-based reading and reporting of SBCE videos.

## Teaser

This diagnostic study develops and evaluates the performance of a convolutional neural network algorithm for review of small bowel capsule endoscopy video in clinical care.

## Introduction

Despite renewed interest in the applicability of capsule endoscopy (CE),^1^ partly owing to pressure on health care systems imposed by the COVID-19 pandemic,^2,3^ the primary clinical use of CE remains the investigation of small bowel (SB) pathology. Besides use in specific clinical situations and visionary proposals,^4^ single-headed CE, which uses 1 camera, remains the “workhorse” of CE. However, irrespective of the capsule manufacturer, experienced health care professionals spend, on average, 50 to 120 minutes reading and reporting on full-length CE recordings.^5^ Reading CE recordings is a tedious task, and although monotonous, it is highly demanding, as it needs dedicated time slots without distractions.^6^

Artificial intelligence is drastically affecting multiple health care domains. Unlike conventional artificial intelligence networks, deep learning consists of several neuronal layers, forming deep neural networks.^7,8^ A deep neural network structure with a significant effect on medical image analysis is the convolutional neural network (CNN).^7,9^ In a study by Ding et al,^10^ a CNN-based algorithm achieved gastroenterologist-level identification of SB diseases and normal variants. However, the interchangeable use of the terms *diagnosis* and *findings* can be confusing, and the criteria used to classify the SB findings (ie, protruding lesions, polyps, and inflammation, which were subsequently used to make a conclusive diagnosis) were not clearly defined. On the other hand, the core of developing a robust computer-aided detection (CADe) system for CE is to detect findings in individual frames.

In this multicenter study, using a large set of SBCE data from procedures performed for clinical care, we developed and evaluated the performance of a CNN-based CADe algorithm, SmartScan, in detecting and classifying 17 types of SB findings based on CE structured terminology (CEST).^11^

## Methods

### Overview of the Study Design

In this diagnostic study, a total of 6097 SBCE examinations performed in 51 Chinese medical centers between January 1, 2012, and June 30, 2020, were retrospectively collected; 272 examinations were excluded due to missing data, leaving data from 5825 SBCE examinations (comprising 295 314 067 images) (eFigure 1 in the Supplement). These data were divided into 2 groups used in the training (training data set) and validation (validation data set) phases of a CADe algorithm (SmartScan) for SBCE findings (eFigures 2 and 3 in the Supplement). The training data set consisted of 2927 SBCE examinations (comprising 148 357 922 images) collected from 29 medical centers. The validation data set consisted of 2898 SBCE examinations (comprising 146 956 145 images), collected from 22 medical centers (eTable 1 in the Supplement). There was no SBCE image overlap between the 2 data sets. There was no overlap between the 29 medical centers where the data for training were collected and the 22 medical centers where the data for validation were collected. Before transfer to the study hub, all personally identifiable information was removed; the SBCE videos were collected in portable nonencrypted external hard drives at the Second Affiliated Hospital of the Third Medical University. Waiver of informed consent from patients was granted for this study because, per the ethical registration and protocol, patients are allowed to give up informed consent. The study was performed following guidelines approved by the medical ethics committee of the Second Affiliated Hospital of the Third Medical University. The study was registered in the Chinese Clinical Trial Registry (CHiCTR2100042455). This study followed the Transparent Reporting of a Multivariable Prediction Model for Individual Prognosis or Diagnosis (TRIPOD) reporting guideline.

### Hardware and Software

Patients were examined with OMOM Capsule Endoscopy System (Chongqing Jinshan Science & Technology Co Ltd). The OMOM Capsule Endoscopy System consists of 3 components: an endoscopic capsule, a data recorder connected with an antenna, and a computer workstation with software for interpretation and reporting of the results. The proprietary reporting software is called VUE. The newer version (VUE Smart), which includes a CNN-based CADe algorithm, SmartScan (trained and validated with the present study), can filter and classify 17 types of SB findings in line with CEST^11^ (eFigure 4 in the Supplement). SmartScan automatically processes every downloaded SBCE video. After SmartScan is completed, reviewers are provided with 2 options: SF, which displays a collection of filtered images with findings and suggested descriptions; and SV, the video mode of images from SF (eFigure 4 in the Supplement). The SF and SV software interfaces display the filtered images by the algorithm.

### Training Phase of SmartScan

SmartScan’s training phase included training data set construction, model training, model tuning, and inference system construction. A total of 2927 SBCE examinations (148 357 922 images) from January 1, 2012, to May 31, 2019, were used in training data set construction. Among the 2927 SBCE examinations, the algorithm training and tuning ratio was about 8:2. After redundant picture scavenging systems processing, 37 089 480 images were obtained, and then 8 endoscopists deleted the images that were not representative. Finally, 757 770 nonrepetitive SBCE images were selected, which included normal images, invalid images, findings-typical images, and findings-atypical images (eFigure 5 in the Supplement). The SmartScan inference system used the cascading decision of the EfficientNet network for primary screening and the YOLO (you only look once) network for secondary screening to construct a step-by-step model to simulate the SBCE reading process and automatically identify abnormal images (eFigure 2 in the Supplement). The EfficientNet network (training data set 1), comprising 608 263 images, is a classification network used for primary screening and classifying all images into 4 types: invalid images, finding-typical images, finding-atypical images, and normal images. The YOLO network (training data set 2), comprising 149 507 images, is a target detection network suitable for recognition of multiple targets and small targets in 1 image. The YOLO network was used for the secondary screening of the categories of normal images and finding-atypical images previously processed by the EfficientNet network. False-positive results of finding-atypical images in the primary screening were reduced; however, the finding-atypical images classified in the primary screening as normal images were screened out to improve the sensitivity of the algorithm in detecting abnormal image recognition. Finally, after the primary and secondary screening, all identified findings were further classified into 17 categories based on CEST in the SmartScan system (eFigure 6 in the Supplement).

### Validation Phase of SmartScan

Eleven specialist gastroenterologists, based in the Second Affiliated Hospital of the Third Medical University, were divided into 2 groups: the experienced SBCE readers group (n = 8; with a mean SBCE reading experience of >200 cases per year) and the expert SBCE readers group (n = 3; senior gastroenterologists with a mean SBCE reading experience of >800 cases per year). Three stages of SBCE reading were scheduled. In stage 1 (January 25 to June 30, 2021), conventional reading (CR) was performed by the experienced SBCE readers group using the older version of VUE software: 2898 SBCE examinations were randomly divided into 8 groups. Each group of examinations (about 362 cases each) was allocated to 1 experienced reader, with data and results recorded. In stage 2 (July 1 to October 1, 2021), SmartScan-assisted reading (SSAR) was performed by the same experienced SBCE readers group that performed the CR, using the new software VUE Smart integrated with the CNN algorithm. The algorithm preread all 2898 examinations and filtered out CEST findings detected for each case, then those cases processed by VUE Smart were randomly divided into 8 groups and allocated to the same 8 experienced readers (approximately 362 cases each), with data and results recorded. In stage 3 (October 10 to December 31, 2021), adjudication on discordant cases only was provided by the expert SBCE readers group. The concordant findings in stage 1 and stage 2, plus the discordant findings adjudicated by the expert SBCE readers group in stage 3 formed the combined agreed comparator. All 11 specialist gastroenterologists were trained together to establish an agreed reading standard, including recognition and classification of 17 CEST findings, reading time recording, and bowel cleanliness grade (eFigure 3 in the Supplement). Both CR and SSAR reading times were recorded to mark the SB section, read SB images, labeling, and report.

### Statistical Analysis

Data analysis was performed from January 25 to December 31, 2021, using SPSS, version 23.0 (IBM Corp). Qualitative indicators were described by frequency table and percentage of composition ratio. Quantitative indicators were described using mean (SD), median (IQR), maximum, and minimum values. The nonparametric Wilcoxon signed-rank test was used for comparing the reading time and number of images in CR and SSAR. A paired χ^2^ test (McNemar test) was performed with the difference of accurate detection rate of finding types between the 2 models. Where the value of the indicators reached 100%, the 95% CI calculation was based on the modified Wald method,^12^ with all *P* values from 2-sided tests and results deemed statistically significant at *P* < .05.

## Results

### Patient Demographics and the Subtypes of CEST Findings

A total of 2898 examinations of SBCE (1765 male participants [60.9%]; mean [SD] age, 49.8 [15.5] years) were included in the validation phase of SmartScan (eTable 2 in the Supplement). The SmartScan system could recognize 17 kinds of subtypes of abnormal SBCE images, including venous structure, nodule, mass or tumor, polyp(s), angioectasia, plaque (red), plaque (white), red spot, abnormal villi, lymphangiectasia, erythematous, edematous, erosion, ulcer, aphtha, blood, and parasite (Figure; eFigure 7 and eTable 3 in the Supplement).

**Figure.**
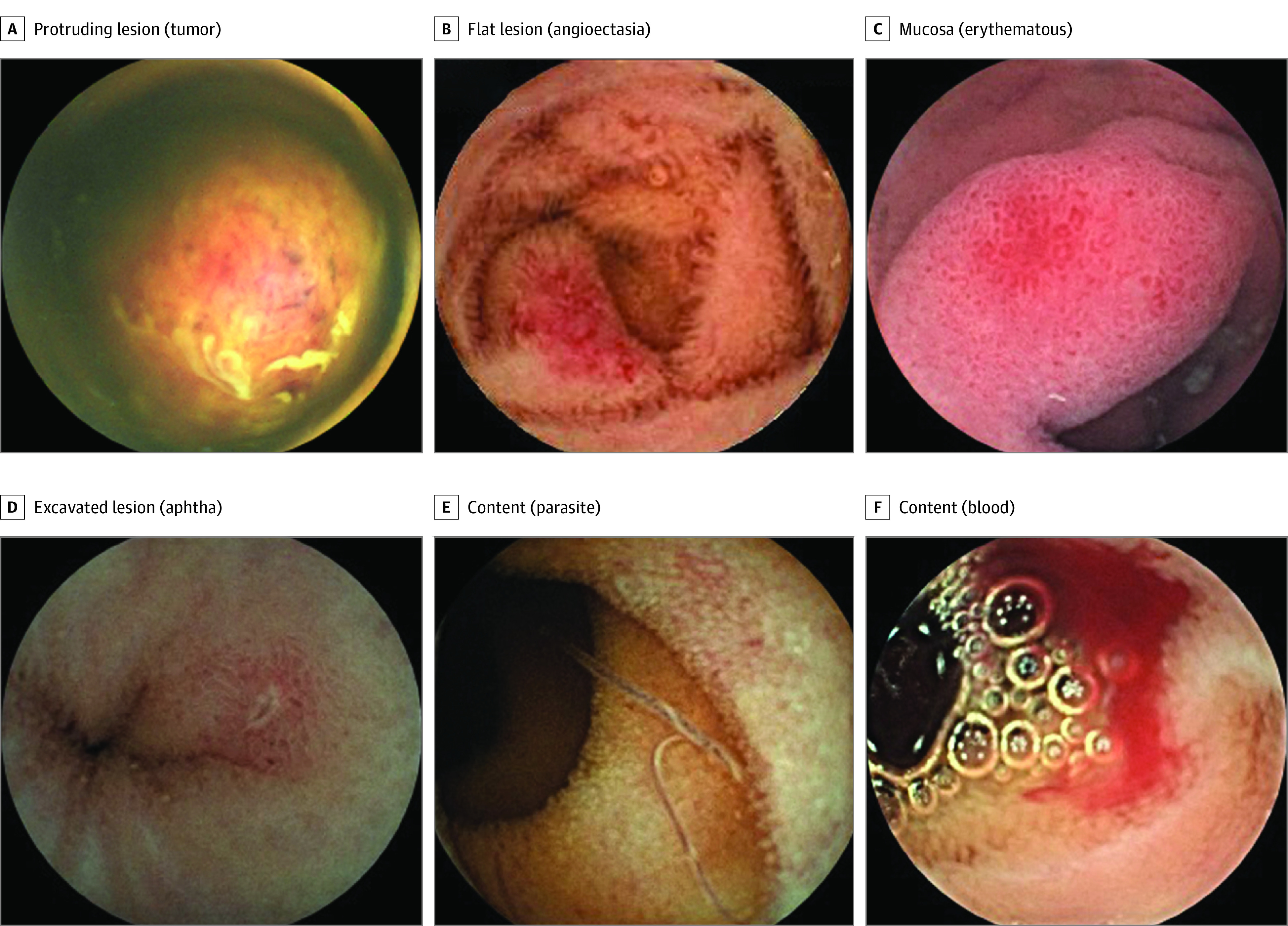
The Typical Findings Detected by SmartScan-Assisted Reading

### Detection Rate of CEST-Based Findings by SSAR

Among 2898 patients, SSAR detected findings in 2298 (79.3%), while CR detected findings in 2048 (70.7%). In a total of 6084 findings, SSAR detected 5834 findings (95.9%; 95.9%; 95% CI, 95.4%-96.4%), while CR detected 4630 findings (76.1%; 95% CI, 75.0%-77.2%) (Table 1). Specifically, based on CEST findings and lesions categories, SSAR achieved a higher detection rate than CR in all CEST categories (Table 2). Among patients with 3 findings or more, the detection rate was significantly higher with SSAR than with than CR, while the detection rate was higher with CR than with SSAR among those with 2 findings or fewer (Table 3). Concerning overall CEST findings, SSAR achieved a 10.7% higher sensitivity than CR (98.8%; 95% CI, 98.3%-99.2% vs 88.1%; 95% CI, 86.7%-89.3%; *P* < .001; eTable 4 in the Supplement). Specifically, SSAR achieved higher sensitivity than CR for all 17 subtypes of findings (Table 4). The comparison of findings missed by SSAR and CR was also an important indicator for evaluating the safety of SSAR. SmartScan-assisted reading missed findings in 28 patients (1.0%) that were detected by CR, while CR missed findings in 278 patients (9.6%) that were detected by SSAR. Overall, SSAR missed 250 of 6084 findings (4.1%), while CR missed 1454 of 6084 findings (23.9%) (eTable 5 in the Supplement). Representative missed findings by CR and SSAR are presented in eFigure 8 in the Supplement. These results suggest that SSAR achieved an overall higher detection rate and missed fewer CEST findings than CR.

**Table 1.** Overall Detection of Findings by Conventional Reading and SmartScan-Assisted Reading

Characteristic	No.	Conventional reading	SmartScan-assisted reading	*P* value	Combined agreed comparator
Patients, No. (%)					
With findings	2898	2048 (70.7)	2298 (79.3)	<.001	2326 (80.3)
Without findings		850 (29.3)	600 (20.7)		572 (19.7)
Small bowel capsule endoscopy findings, No. (%)	6084	4630 (76.1)	5834 (95.9)	<.001	6084 (100.00)

**Table 2.** Detection Rate of Subtypes of Findings by Conventional Readings and SmartScan-Assisted Reading

Category	No. (%) (N = 2898)		χ^2^ value	*P* value
	Conventional readings	SmartScan-assisted reading		
Protruding lesions				
Venous structure	311 (10.7)	414 (14.3)	16.725	<.001
Nodule	545 (18.8)	676 (23.3)	17.806	<.001
Mass or tumor	104 (3.6)	131 (4.5)	3.233	.07
Polyp(s)	254 (8.8)	327 (11.3)	10.194	.001
Flat lesions				
Angioectasia	75 (2.6)	96 (3.3)	2.657	.10
Plaque (red)	236 (8.1)	296 (10.2)	7.451	.006
Plaque (white)	434 (15.0)	562 (19.4)	19.863	.001
Red spot	232 (8.0)	309 (10.7)	12.088	.001
Abnormal villi	370 (12.8)	449 (15.5)	8.874	.003
Mucosa				
Lymphangiectasia	759 (26.2)	998 (34.4)	46.653	<.001
Erythematous	324 (11.2)	395 (13.6)	8.004	.005
Edematous	136 (4.7)	166 (5.7)	3.144	.08
Excavated lesions				
Erosion	266 (9.2)	336 (11.6)	9.083	.003
Ulcer	267 (9.2)	293 (10.1)	1.336	.25
Aphtha	61 (2.1)	84 (2.9)	3.742	.05
Content				
Blood	189 (6.5)	228 (7.9)	3.930	.047
Parasite	67 (2.3)	74 (2.6)	0.356	.55

**Table 3.** Detection of Multiple Findings Types by Conventional Reading and SmartScan-Assisted Reading

Type, No.	Patients with multiple findings, No. (%) (N = 2898)		*P* value	Combined agreed comparator, No. (%) (N = 2898)
	Conventional readings	SmartScan-assisted reading		
≤2	2172 (75.0)	1878 (64.8)	<.001	1819 (62.8)
3	420 (14.5)	522 (18.0)	<.001	535 (18.5)
4	163 (5.6)	269 (9.3)	<.001	281 (9.7)
≥5	143 (4.9)	229 (7.9)	<.001	263 (9.1)

**Table 4.** Comparison of 17 Types of Findings by Conventional Reading and SmartScan-Assisted Reading

Index	% (95% CI)		*P* value
	Conventional readings	SmartScan-assisted reading	
**Protruding lesions**			
Venous structure			
Sensitivity	73.7 (69.3-77.7)	98.1 (96.3-99.0)	<.001
Specificity	100 (99.8-100)	100 (99.8-100)	≥.99
Nodule			
Sensitivity	78.2 (75.0-81.1)	97.0 (95.4-98.0)	<.001
Specificity	100 (99.8-100)	100 (99.8-100)	≥.99
Mass or tumor			
Sensitivity	75.4 (67.6-81.8)	94.9 (89.9-97.5)	.003
Specificity	100 (99.8-100)	100 (99.8-100)	≥.99
Polyp(s)			
Sensitivity	73.8 (69.0-78.2)	95.1 (92.2-96.9)	<.001
Specificity	100 (99.8-100)	100 (99.8-100)	≥.99
**Flat lesions**			
Angioectasia			
Sensitivity	75.0 (65.7-82.5)	96.0 (90.2-98.4)	<.001
Specificity	100 (99.8-100)	100 (99.8-100)	≥.99
Plaque (red)			
Sensitivity	74.9 (69.9-79.4)	94.0 (90.8-96.1)	<.001
Specificity	100 (99.8-100)	100 (99.8-100)	≥.99
Plaque (white)			
Sensitivity	73.3 (69.6-76.7)	94.9 (92.9-96.4)	<.001
Specificity	100 (99.8-100)	100 (99.8-100)	≥.99
Red spot			
Sensitivity	72.3 (67.1-76.9)	96.3 (93.6-97.9)	<.001
Specificity	100 (99.8-100)	100 (99.8-100)	≥.99
Abnormal villi			
Sensitivity	78.4 (74.5-81.9)	95.1 (92.8-96.7)	<.001
Specificity	100 (99.8-100)	100 (99.8-100)	≥.99
**Mucosa**			
Lymphangiectasia			
Sensitivity	74.4 (71.7-77.0)	97.8 (96.8-98.6)	<.001
Specificity	100 (99.8-100)	100 (99.8-100)	≥.99
Erythematous			
Sensitivity	77.9 (73.7-84.6)	95.0 (92.4-96.7)	<.001
Specificity	100 (99.8-100)	100 (99.8-100)	≥.99

### Efficiency of SSAR in Reading SBCE Video to Detect Intestinal Findings

With SSAR, the mean (SD) number of images per SBCE video was reduced to 779.2 (337.2) (median, 861; IQR, 502-1044), compared with 27 910.8 (12 882.9) (median, 26 277; IQR, 19 218-35 673) with CR, for a mean (SD) reduction rate of 96.1% (4.3%) (eTable 6 in the Supplement). In addition, the mean (SD) reading time with SSAR was 5.4 (1.5) minutes (median, 5 minutes; IQR, 4-6 minutes), compared with 51.4 (11.6) minutes (median, 50 minutes [IQR, 43-58 minutes]) with CR, for a mean (SD) reduction rate of 89.3% (3.1%).

### Patient Diagnoses

Clinical diagnoses presented in the study were based on the SBCE findings of the combined agreed comparator. Among 2898 SBCEs, 1647 were confirmed as abnormal, providing a diagnostic yield of 56.8%, not including normal variants, such as lymphangiectasias, white plaques, or venous structures. In these 1647 abnormal SBCEs, 2169 diagnoses were made in total. Inflammation of all grades, was found in 31.2% of patients (905 of 2898), followed by vascular abnormalities (17.3% [501 of 2898]) and neoplasia (15.5% [450 of 2898]) (eTable 7 in the Supplement).

## Discussion

In this study, 5825 SBCEs were collected to train and subsequently validate SmartScan, a proprietary CNN-based CADe algorithm. Unlike in previous studies,^10^ all SBCEs in this study were based on clinically established pathways (or recommendations) to investigate SB abnormalities, including SB bleeding, iron deficiency anemia, abdominal pain or diarrhea, and unexplained weight loss. In the validation phase, which included 2898 patients, SSAR achieved an overall higher detection rate for CEST findings compared with CR (79.3% vs 70.7%); from a total of 6084 SB findings, SSAR detected 95.9%, significantly higher compared with CR (76.1%). Furthermore, SSAR achieved overall higher sensitivity for the 17 CEST subtypes of findings compared with CR (98.8% vs 88.1%). The mean (SD) number of images requiring review per SBCE video was reduced to 779.2 (337.2) with SSAR, compared with 27 910.8 (12 882.9) images with CR; the mean (SD) reading time with SSAR was shortened to 5.4 (1.5) minutes, compared with 51.4 (11.6) minutes with CR, for a mean (SD) reduction rate of 89.3% (3.1%).

Overall, SSAR showed superior performance compared with CR while significantly reducing reading times. However, 1454 findings (23.9%) were missed by CR and 250 findings (4.1%) were missed by SSAR. Although the rate of findings missed by CR is consistent with the relevant literature,^13^ findings missed by SSAR were associated with various reasons such as atypical image, a limited number of captured frames of specific findings, and/or overall image quality. Currently, reporting SBCE is a task of single or paired reviewers (prereading or reading approach) and constrained by human error.^14^ Some works suggest that physicians’ performance is disappointing^15,16^ and that the reporting accuracy in SBCE declines after reading a single capsule study.^17^ Because incomplete visualization of the SB in terms of coverage and/or image quality affects mainly the detection and reporting of neoplastic lesions,^18^ it becomes evident that adjustable frame rate and higher-resolution images are needed. However, although these alterations can improve CNN-based CADe performance, they cannot resolve the issue of human error in reporting or the low level of interobserver agreement, regardless of the readers’ experience.^16^

After 2 decades of clinical use, CE claims a prime diagnostic role in the SB.^19^ However, it still has several significant limitations such as the use, type, and timing of bowel preparation^20^; the time required for conventional SBCE reading; and the overall suboptimal interobserver agreement between readers.^16^ Moreover, the clinical relevance of any findings is vital for a conclusive diagnosis. For instance, a typical angiectasia is regarded as highly relevant in the clinical setting of SB bleeding but perceived as not relevant in the setting of suspected Crohn disease.^21^ In 2005, CEST was introduced as a first attempt to structure terminology in CE and assist unification of reporting.^11,22^ Since then, complementary and well-received studies have been published attempting to revamp and refine the CEST nomenclature^23,24^ and eventually guide clinicians in assessing the clinical relevance of findings in SBCE.^21^ Eventually, to develop an accurate, reliable, and clinically applicable artificial intelligence software, all this experience should be translated into developing a CNN-based algorithm by rooting out all human-related confounders.

At the initial stage, any CADe system must detect all findings, as this is its first critical test that will allow its broader implementation into clinical practice. Thereafter, appropriate categorization and clustering of provided images and findings by human readers are used to name these findings (pathology) and/or deliver a diagnosis for each individual case. It has been shown that higher accuracy and better interobserver agreement can be achieved by amassing more CE reporting experience and using consensus and CEST.^22^ In the validation data set of our study, consisting of 2898 SBCEs, 2326 (80.3%) had SB findings, which were further evaluated based on the clinical setting (ie, indications). Subsequently, 1647 SBCEs (56.8%) were considered abnormal, similar to the overall 59.4% detection rate in one meta-analysis.^13^ In 679 SBCEs (23.4%), the detected findings were considered of no clinical relevance and were therefore normal.

The last few years saw artificial intelligence entering a new level of clinical application in gastrointestinal endoscopy. Despite being a prime target for applying artificial intelligence algorithms, CE was the last to see a commercially available solution,^10^ available only recently (eTable 8 in the Supplement). Automatic hemorrhage detection with video CE was the first issue that caught information technology scientists’ interest, whereas lesion detection, reduction of video review time, and quality enhancement were the subsequent focus.^25^ In 2014, a simple yet effective approach allowing automatic detection of all types of abnormalities in CE was presented.^26^ The proposed software method, based on color pattern recognition, outperformed previous state-of-the-art approaches. Moreover, it reported robust results in the presence of luminal contents and could detect even tiny lesions. However, just like previous attempts, it was limited by the actual small number of images included. It has become apparent, therefore, that large data sets are desirable.^27^ However, their annotation has always been a barrier, as it requires a considerable amount of effort from expert CE reviewers (usually more than 1 reviewer per data set to enable assessment of interobserver agreement).^28,29,30,31^

### Limitations

This study has some limitations. First, a solid reference standard (in this study, the combined agreed comparator) was lacking in the validation phase. A more appropriate reference standard should have been a committee of expert SBCE readers (with reading experience of >3000 cases each), who will read each video individually at a predefined speed^6^ using agreed CEST and record all relevant findings against which any CNN CADe should be checked. Admittedly, this task cannot be achieved easily and requires time and effort from clinicians already overburdened by daily duties and the effects of the recent COVID-19 pandemic.^32^ Moreover, one could argue that an expert committee will be accepted as a criterion standard with some reservations, considering associated human factors and quality assessment definitions.^15,33^ For this study, because of the number of SBCEs included, the combined agreed comparator was considered the best possible alternative to a criterion standard. Second, SBCE data were retrospectively collected from several regions in China. Although every attempt was made to involve several different regions to ensure clinical diversity, the results may not be generalizable to other world areas, Therefore, well-constructed prospective studies with sizeable data sets from different world regions are needed to evaluate the clinical performance of SmartScan further. Third, SmartScan’s findings miss rate of 4.1% in the validation data set, seems far from the required optimal or even acceptable miss rate for a CNN-based software. However, this study was performed with data obtained from earlier versions of the OMOM Capsule Endoscopy System.

## Conclusions

The findings of this study suggest that SmartScan is associated with an increased detection rate of SB findings and reduced SBCE reading times. This CNN-based software was developed with the CEST in mind and main aim to allow a reproducible classification of its sensitivity results in further prospective studies.

## Notes

Supplement. **eFigure 1.** Geographical Distribution of Centers and Included Cases **eFigure 2.** Study Flow Chart of Training Phase **eFigure 3.** Study Flow Chart of Validation Phase **eFigure 4.** Capsule Endoscopy System and Software **eFigure 5.** Normal Images, Invalid Images, Findings-Typical Images, and Findings-Atypical Images **eFigure 6.** Flow Chart of *SmartScan* Inference System **eFigure 7.** The 17 Findings Detected by *SmartScan* Assisted Reading (SSAR) **eFigure 8.** Missed Findings by Conventional Reading (CR) and *SmartScan*-Assisted Reading (SSAR) **eTable 1.** Detailed Information of Patients in the Validation Set **eTable 2.** Demographics of Enrolled Subjects (N = 2898) **eTable 3.** Comparison of 17 Types of Findings by Conventional Reading (CR) and *SmartScan* Assisted Reading (SSAR) **eTable 4.** Sensitivity of Findings in Patients Conventional Reading (CR) and *SmartScan* Assisted Reading (SSAR) (N = 2898) **eTable 5.** Missed Findings by Conventional Reading (CR) and *SmartScan* Assisted Reading (SSAR) **eTable 6.** Reading Time and Number of Images by Conventional Reading (CR) and *SmartScan* Assisted Reading (SSAR) **eTable 7.** Clinical Diagnosis Based on the Findings of Combined Agreed Comparator **eTable 8.** Different Studies of Artificial Intelligence Application in Capsule Endoscopy
