# Supplementary material for: Development and Validation of an Artificial Intelligence Model for Small Bowel Capsule Endoscopy Video Review
Source: JAMA Netw Open. 2022 Jul 14;5(7):e2221992. doi: 10.1001/jamanetworkopen.2022.21992 (PMC9284338; doi:10.1001/jamanetworkopen.2022.21992)
Supplement: Supplement. — eFigure 1. Geographical Distribution of Centers and Included Cases eFigure 2. Study Flow Chart of Training Phase eFigure 3. Study Flow Chart of Validation Phase eFigure 4. Capsule Endoscopy System and Software eFigure 5. Normal Images, Invalid Images, Findings-Typical Images, and Findings-Atypical Images eFigure 6. Flow Chart of SmartScan Inference System eFigure 7. The 17 Findings Detected by SmartScan Assisted Reading (SSAR) eFigure 8. Missed Findings by Conventional Reading (CR) and SmartScan-Assisted Reading (SSAR) eTable 1. Detailed Information of Patients in the Validation Set eTable 2. Demographics of Enrolled Subjects (N = 2898) eTable 3. Comparison of 17 Types of Findings by Conventional Reading (CR) and SmartScan Assisted Reading (SSAR) eTable 4. Sensitivity of Findings in Patients Conventional Reading (CR) and SmartScan Assisted Reading (SSAR) (N = 2898) eTable 5. Missed Findings by Conventional Reading (CR) and SmartScan Assisted Reading (SSAR) eTable 6. Reading Time and Number of Images by Conventional Reading (CR) and SmartScan Assisted Reading (SSAR) eTable 7. Clinical Diagnosis Based on the Findings of Combined Agreed Comparator eTable 8. Different Studies of Artificial Intelligence Application in Capsule Endoscopy [file jamanetwopen-e2221992-s001.pdf]

# Supplementary Online Content

Xie X, Xiao YF, Zhao XY, et al. Development and validation of an artificial intelligence model for small bowel capsule endoscopy video review. *JAMA Netw Open.* 2022;5(7):e2221992. doi:10.1001/jamanetworkopen.2022.21992

**eFigure 1.** Geographical Distribution of Centers and Included Cases
**eFigure 2.** Study Flow Chart of Training Phase
**eFigure 3.** Study Flow Chart of Validation Phase
**eFigure 4.** Capsule Endoscopy System and Software
**eFigure 5.** Normal Images, Invalid Images, Findings-Typical Images, and Findings-Atypical Images
**eFigure 6.** Flow Chart of *SmartScan* Inference System
**eFigure 7.** The 17 Findings Detected by *SmartScan* Assisted Reading (SSAR)
**eFigure 8.** Missed Findings by Conventional Reading (CR) and *SmartScan*-Assisted Reading (SSAR)
**eTable 1.** Detailed Information of Patients in the Validation Set
**eTable 2.** Demographics of Enrolled Subjects (N=2898)
**eTable 3.** Comparison of 17 Types of Findings by Conventional Reading (CR) and *SmartScan* Assisted Reading (SSAR)
**eTable 4.** Sensitivity of Findings in Patients Conventional Reading (CR) and *SmartScan* Assisted Reading (SSAR) (N = 2898)
**eTable 5.** Missed Findings by Conventional Reading (CR) and *SmartScan* Assisted Reading (SSAR)
**eTable 6.** Reading Time and Number of Images by Conventional Reading (CR) and *SmartScan* Assisted Reading (SSAR)
**eTable 7.** Clinical Diagnosis Based on the Findings of Combined Agreed Comparator
**eTable 8.** Different Studies of Artificial Intelligence Application in Capsule Endoscopy

This supplementary material has been provided by the authors to give readers additional information about their work.

© 2022 Xie X et al. *JAMA Network Open.*

**eFigure 1.** Geographical Distribution of Centers and Included Cases

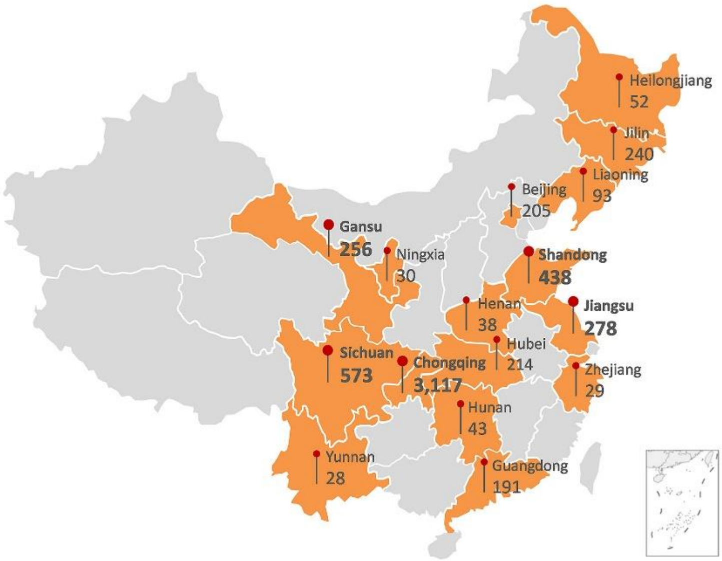


| No. | Province | Cases |
|---|---|---|
| **1** | **Chongqing** | **3,117** |
| **2** | **Sichuan** | **573** |
| **3** | **Shandong** | **438** |
| **4** | **Jiangsu** | **278** |
| **5** | **Gansu** | **256** |
| 6 | Jilin | 240 |
| 7 | Hubei | 214 |
| 8 | Beijing | 205 |
| 9 | Guangdong | 191 |
| 10 | Liaoning | 93 |
| 11 | Heilongjiang | 52 |
| 12 | Hunan | 43 |
| 13 | Henan | 38 |
| 14 | Ningxia | 30 |
| 15 | Zhejiang | 29 |
| 16 | Yunnan | 28 |
| | Total: | 5,825 |

© 2022 Xie X et al. *JAMA Network Open.*

**eFigure 2.** Study Flow Chart of Training Phase

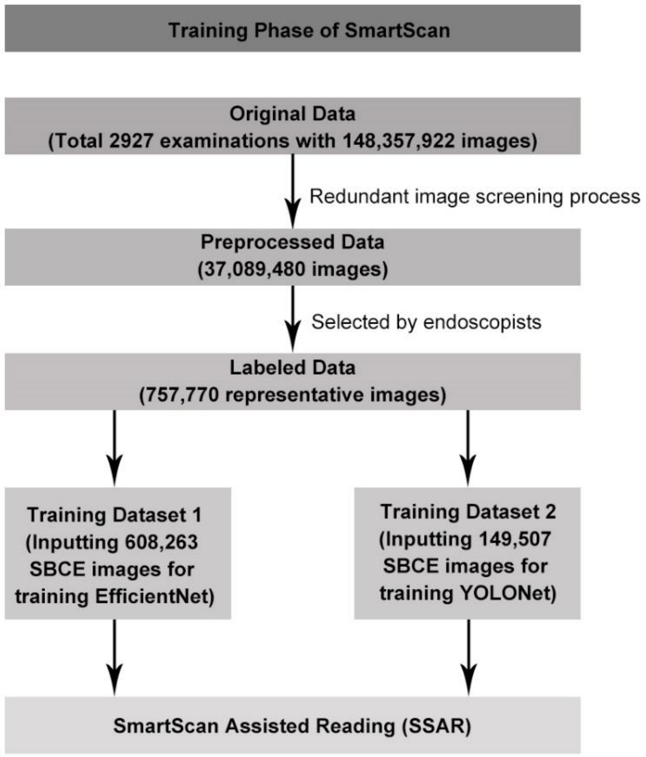


© 2022 Xie X et al. *JAMA Network Open.*

**eFigure 3.** Study Flow Chart of Validation Phase

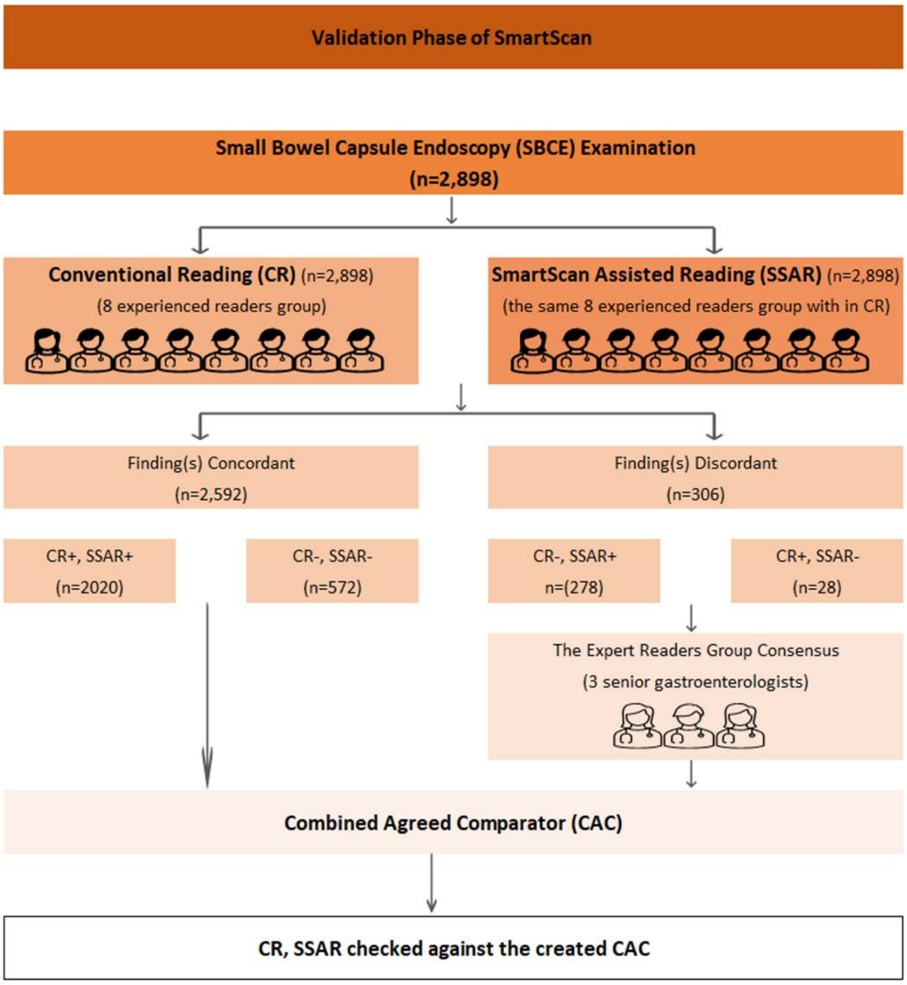


© 2022 Xie X et al. *JAMA Network Open.*

**eFigure 4.** Capsule Endoscopy System and Software. (A) Hardware of Capsule Endoscopy; (B) Main Interface of software; (C) “SmartView” & ”SmartFindings”.

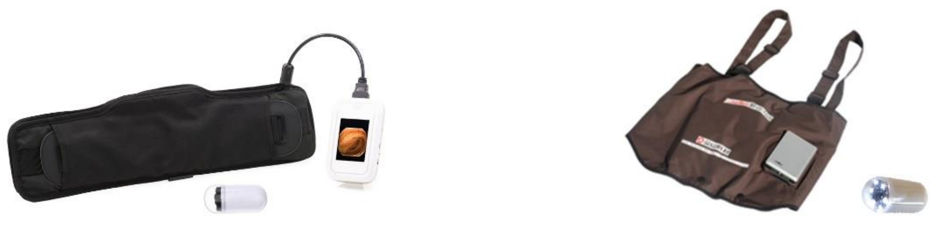

(JS-MEC-I) (JS-MEC-II)

A. Hardware of Capsule Endoscopy

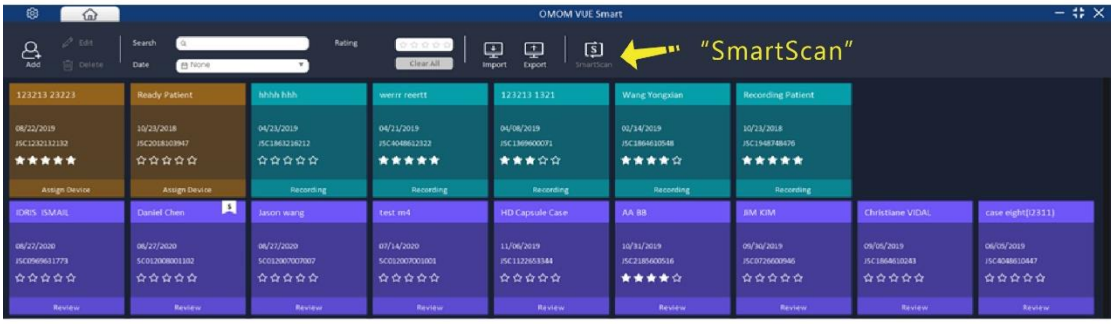


B. Main Interface of Software

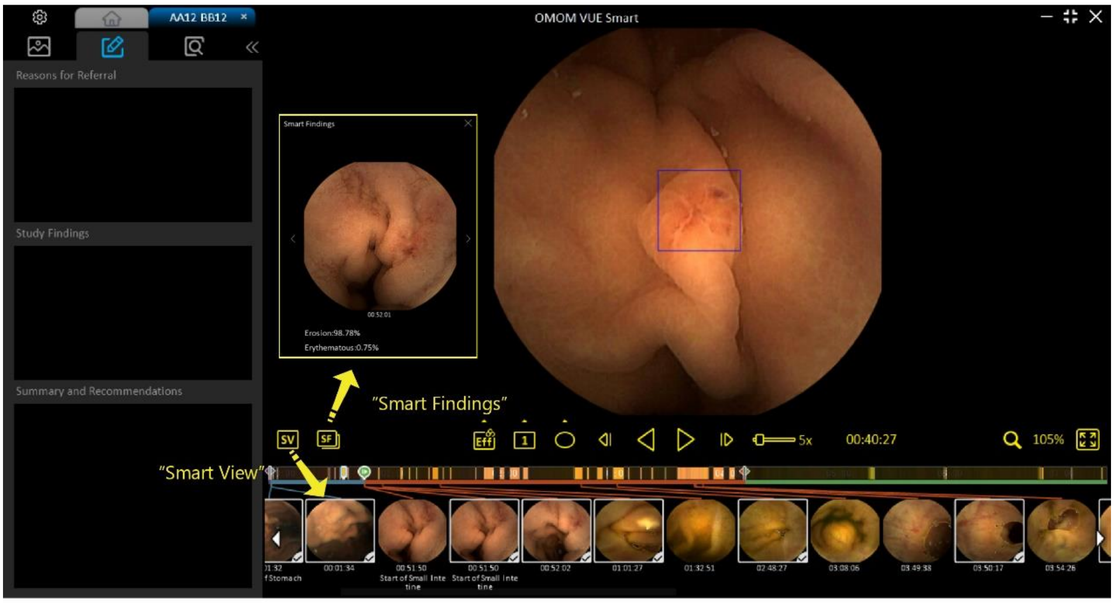


C. “SmartView” &”SmartFinding”

© 2022 Xie X et al. *JAMA Network Open.*

**eFigure 5.** Normal Images, Invalid Images, Findings-Typical Images, and Findings-Atypical Images

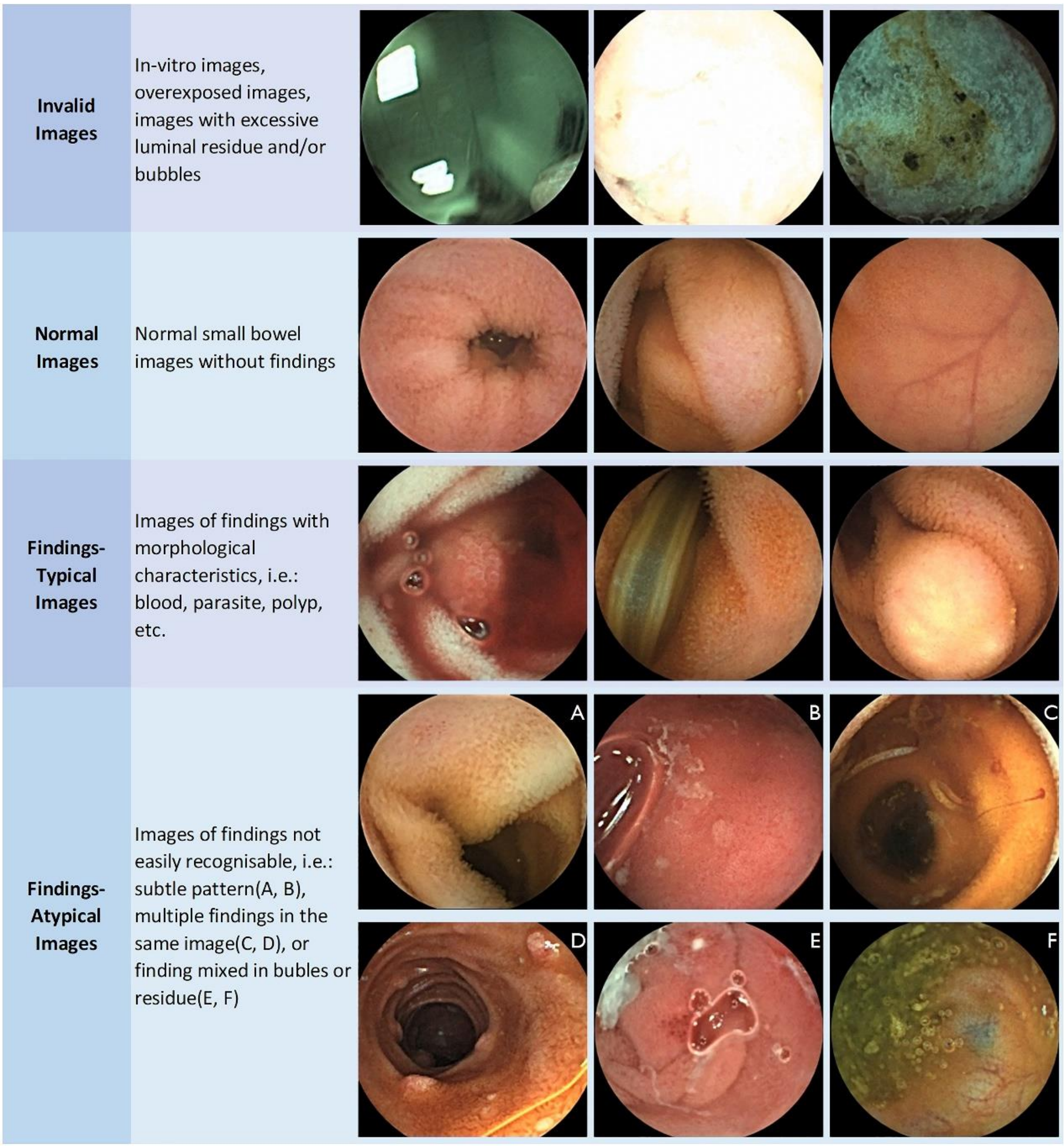


© 2022 Xie X et al. *JAMA Network Open.*

**eFigure 6.** Flow Chart of *SmartScan* Inference System. (A) Flow Chart of *SmartScan* Inference System; (B) EfficientNet Network; (C) YOLO Network.

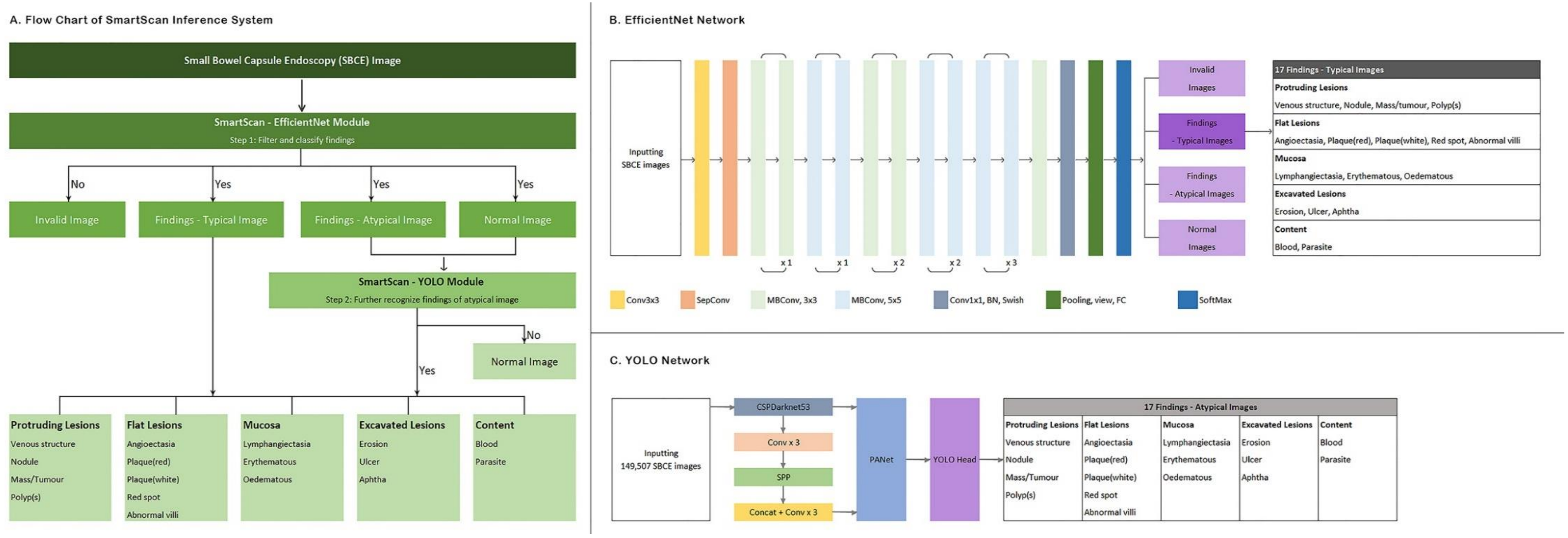


© 2022 Xie X et al. *JAMA Network Open*.

**eFigure 7.** The 17 Findings Detected by *SmartScan* Assisted Reading (SSAR)

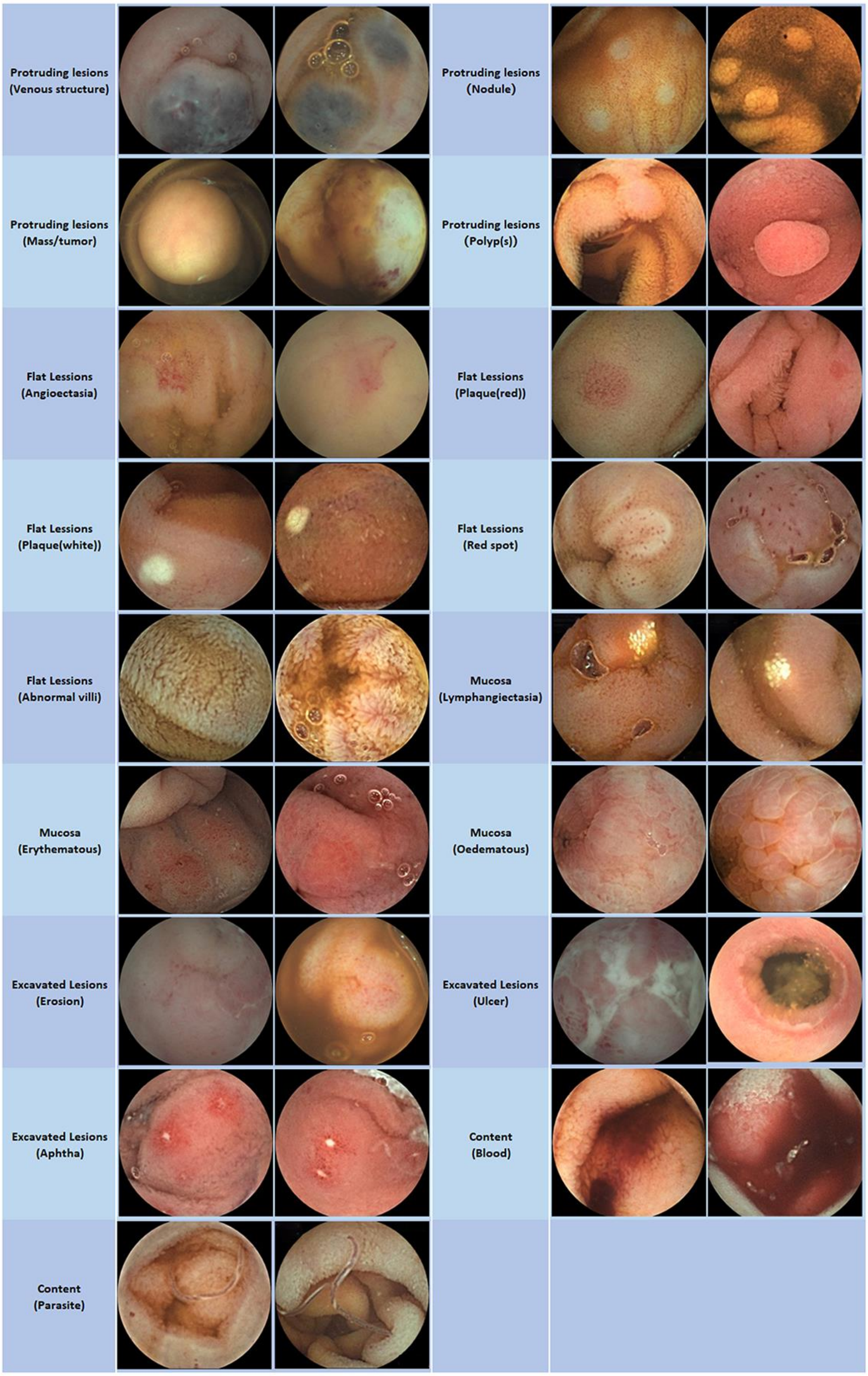


© 2022 Xie X et al. *JAMA Network Open.*

**eFigure 8.** Missed Findings by Conventional Reading (CR) and *SmartScan*-Assisted Reading (SSAR)

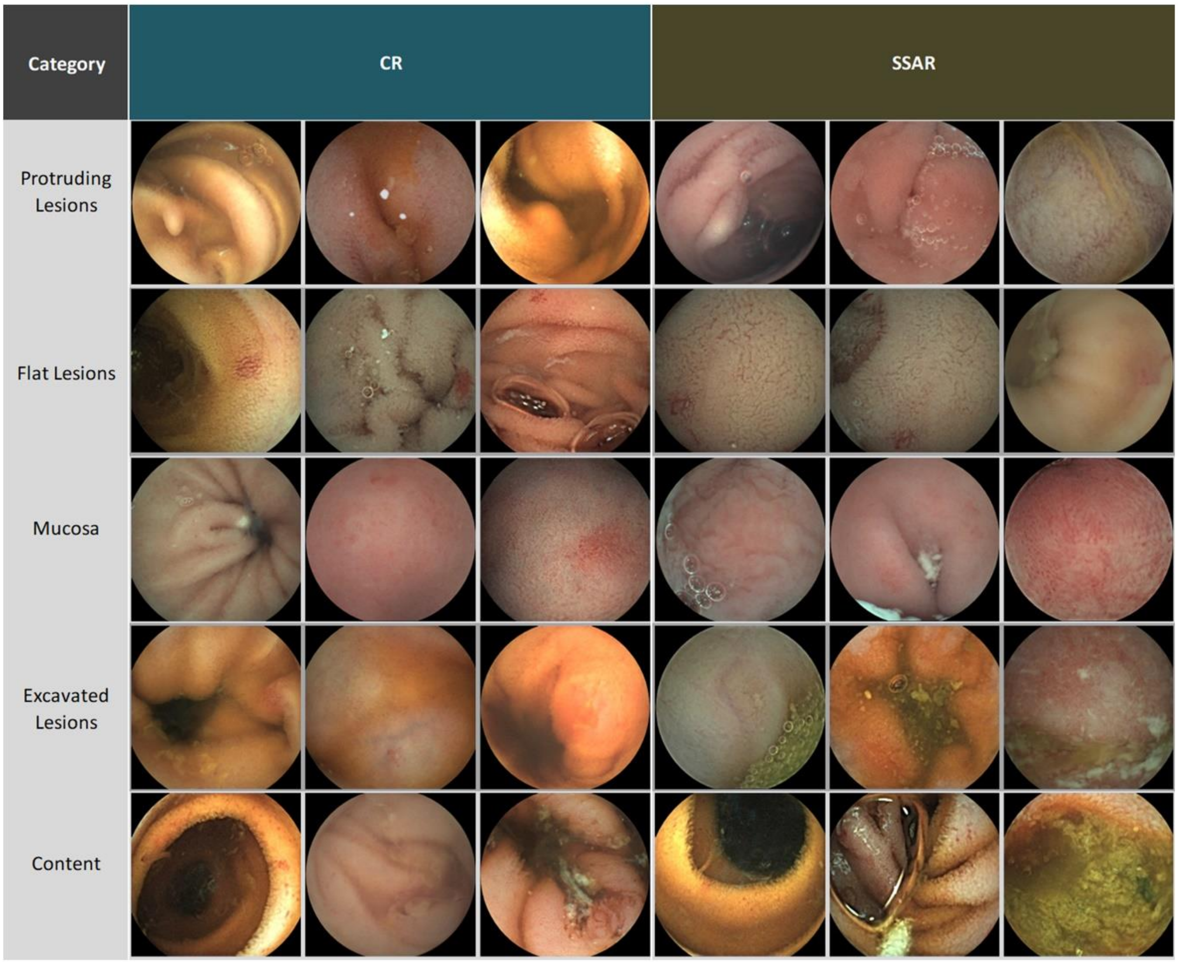


© 2022 Xie X et al. *JAMA Network Open.*

**eTable 1.** Detailed Information of Patients in the Validation Set

| Name of Hospital | Number of included studies | Number of patients enrolled | Number of excluded cases |
|---|---|---|---|
| Chengdu Military Region General Hospital | 74 | 80 | 6 |
| Henan Provincial People's Hospital | 38 | 40 | 2 |
| Heilongjiang Provincial People's Hospital | 27 | 29 | 2 |
| Hubei Provincial People's Hospital | 190 | 192 | 2 |
| West China Hospital | 48 | 49 | 1 |
| Bahun First Hospital of Jilin University | 179 | 179 | 0 |
| PLA Air Force General Hospital | 30 | 31 | 1 |
| The Second Affiliated Hospital of Lanzhou University | 32 | 34 | 2 |
| The First Affiliated Hospital of Lanzhou University | 57 | 70 | 13 |
| Daping Hospital, Army Military Medical University | 24 | 24 | 0 |
| Xinqiao Hospital, Army Military Medical University | 994 | 1023 | 29 |
| Nanchuan hospital | 208 | 223 | 15 |
| Southern Hospital | 173 | 174 | 1 |
| General Hospital of Nanjing Military Region | 200 | 272 | 72 |
| Ningxia Kangyuan anorectal Hospital | 11 | 11 | 0 |
| Ningxia Medical University General Hospital | 19 | 19 | 0 |
| Qilu Hospital of Shandong Province, China | 397 | 456 | 59 |
| Sichuan Provincial People's Hospital | 28 | 29 | 1 |
| Hospital of Southwest Medical University | 49 | 56 | 7 |
| The first people's Hospital of Yunnan Province | 23 | 27 | 4 |
| The Second Affiliated Hospital of Zhejiang University | 29 | 29 | 0 |
| The Second Affiliated Hospital of Chongqing Medical University | 68 | 76 | 8 |
| Total | 2898 | 3123 | 225 |

© 2022 Xie X et al. *JAMA Network Open.*

**eTable 2.** Demographics of Enrolled Subjects (N=2898)

| | | |
|---|---|---|
| **Gender, No. (%)** | Female | 1,133(39.1) |
| | Male | 1,765(60.9) |
| **Age (y)** | Mean ±SD | 49.76 ±15.49 |
| | Med (P25-P75) | 50(39-62) |
| | Med (Mix-Max) | 50(5-93) |
| **SB cleanliness, No. (%)** | Good | 638(22.0) |
| | Medium | 1,584(54.7) |
| | Poor | 676(23.3) |
| **SB completion rate, No. (%)** | No | 319(11.01) |
| | Yes | 2,579(88.99) |
| **Stomach transit time (min)** | Mean ±SD | 53.13±58.85 |
| | Med (P25-P75) | 33.33(13.43-72.63) |
| | Med (Mix-Max) | 33.33(0.22-827.75) |
| **SB Transit Time (min)** | Mean ±SD | 299.89 ±128.41 |
| | Med (P25-P75) | 276.69(221.0-365.98) |
| | Med (Mix-Max) | 276.69(9.82-934.68) |
| **Capsule working time (min)** | Mean ±SD | 579.27 ±137.07 |
| | Med (P25-P75) | 600.0(475.53-668.08) |
| | Med (Mix-Max) | 600.0(35.35-1,054.50) |

© 2022 Xie X et al. *JAMA Network Open.*

**eTable 3.** Comparison of 17 Types of Findings by Conventional Reading (CR) and *SmartScan* Assisted Reading (SSAR)

| | |
|---|---|
| **Protruding Lesions** | Venous structure |
| | Nodule |
| | Mass/Tumour |
| | Polyp(s) |
| **Flat Lesions** | Angioectasia |
| | Plaque(red) |
| | Plaque(white) |
| | Red spot |
| | Abnormal villi |
| **Mucosa** | Lymphangiectasia |
| | Erythematous |
| | Oedematous |
| **Excavated Lesions** | Erosion |
| | Ulcer |
| | Aphtha |
| **Content** | Blood |
| | Parasite |

© 2022 Xie X et al. *JAMA Network Open.*

**eTable 4.** Sensitivity of Findings in Patients Conventional Reading (CR) and *SmartScan* Assisted Reading (SSAR) (N = 2898)

| | CR % (95% CI) | SSAR % (95% CI) | P value |
|---|---|---|---|
| **Sens** | 88.05 (86.67-89.30) | 98.80 (98.27-99.17) | <0.001 |
| **Spec** | 100.00 (99.20-100.00) | 100.00 (99.20-100.00) | 1.000 |
| **+PV** | 100.00 (99.77-100.00) | 100.00 (99.79-100.00) | 1.000 |
| **-PV** | 67.29 (64.07-70.36) | 95.33 (93.31-96.78) | <0.001 |

© 2022 Xie X et al. *JAMA Network Open.*

**eTable 5.** Missed Findings by Conventional Reading (CR) and *SmartScan* Assisted Reading (SSAR)

| **Findings** | | CR, No. | SSAR No. |
|---|---|---|---|
| **Protruding Lesions** | Venous structure | 111 | 8 |
| | Nodule | 152 | 21 |
| | Mass/tumour | 34 | 7 |
| | Polyp(s) | 90 | 17 |
| **Flat Lesions** | Angioectasia | 25 | 4 |
| | Plaque(red) | 79 | 19 |
| | Plaque(white) | 158 | 30 |
| | Red spot | 89 | 12 |
| | Abnormal villi | 102 | 23 |
| **Mucosa** | Lymphangiectasia | 261 | 22 |
| | Erythematous | 92 | 21 |
| | Oedematous | 44 | 14 |
| **Excavated Lesions** | Erosion | 89 | 19 |
| | Ulcer | 45 | 19 |
| | Aphtha | 26 | 3 |
| **Content** | Blood | 47 | 8 |
| | Parasite | 10 | 3 |

© 2022 Xie X et al. *JAMA Network Open.*

**eTable 6.** Reading Time and Number of Images by Conventional Reading (CR) and *SmartScan* Assisted Reading (SSAR)

| | | CR | SSAR | P value | Difference between two readings |
|---|---|---|---|---|---|
| SBCE Reading time (min) | Mean (SD) | 51.42(11.60) | 5.37(1.51) | | 46.05(11.16) |
| | Med (IQR) | 50 (43-58) | 5 (4-6) | <0.001 [a] | 45 (38-53) |
| | Med (range) | 50 (30-130) | 5 (3-12) | | 45 (25-123) |
| Number of SBCE Images | Mean (SD) | 27,910.83(12,882.89) | 779.17(337.18) | | 27,131.66(12,888.95) |
| | Med (IQR) | 26,277 (19,218-35,673) | 861 (502-1,044) | <0.001 [b] | 25,495 (18,398-34,905) |
| | Med (range) | 26,277 (860.0-81,907) | 861 (101-1,554) | | 25,495 (350-81,789) |

a Comparison of reading time between CR and SSAR, non-parametric paired Wilcoxon rank sum test： Z=46.629，P<0.001

b Comparison of the number of pictures between CR and SSAR, non-parametric paired Wilcoxon rank sum test： Z=46.625， P<0.001

© 2022 Xie X et al. *JAMA Network Open.*

**eTable 7.** Clinical Diagnosis Based on the Findings of Combined Agreed Comparator

| | | n | % |
|---|---|---|---|
| **Overall diagnosis (n=2,898)** | Abnormal | 1647 | 56.83 |
| | Normal | 1251 | 43.17 |
| **Abnormal Diagnosis (%, cases/2,898)** | Inflammation- all grades | 905 | 31.23 |
| | Bleeding | 236 | 8.14 |
| | Neoplasia | 450 | 15.53 |
| | Infection | 77 | 2.66 |
| | Vascular abnormalities | 501 | 17.29 |
| | Total | 2,169 | - |

© 2022 Xie X et al. *JAMA Network Open.*

**eTable 8.** Different Studies of Artificial Intelligence Application in Capsule Endoscopy

| | | Present Study | Hou XH, et al. | Hiroaki S, et al. |
|---|---|---|---|---|
| Learning Model Type | | CNN | CNN | CNN |
| Center Number (n) | | 51 | 77 | 3 |
| Total Image (n) | | 295,314,067 | 113,426,569 | 48091 |
| Total Patients (n) | | 5825 | 6970 | 385 |
| Lesion types (n) | | 17 | >10 | 5 |
| Training Dataset | | | | |
| | Images | 148,357,922 | 158,235 | 30,584 |
| | Patients | 2927 | 1970 | 292 |
| Evaluation Dataset | | | | |
| | Images | 146,956,145 | 113,268,334 | 17,507 |
| | Patients | 2898 | 5000 | 93 |
| Reading Time in one case(min) | | | | |
| | Conventional reading | 51.42±11.60 | 96.6 ± 22.53 | Unknown |
| | AI reading | 5.37 ±1.51 | 5.9±2.23 | Unknown |
| Reduced Images in one case(n) | | | | |
| | Conventional reading | 27,910.83±12,882.89 | 22,654 | Unknown |
| | AI reading | 779.17±337.18 | 578 | Unknown |
| Sensitivity%,95% CI | | 98.80(98.27-99.17) | 99.90 (99.74-99.97) | 90.7 (90.0-91.4) |
| Specificity%,95% CI | | 100.0(99.20-100.00) | 100(99.72-100.00) | 79.8 (79.0-80.6) |

© 2022 Xie X et al. *JAMA Network Open.*
